# La radiothérapie du cancer de l'endomètre: expérience de l'institut national d'oncologie à propos de 52 cas

**DOI:** 10.11604/pamj.2016.23.144.4772

**Published:** 2016-03-30

**Authors:** Imane Mezouri, Soufiane Berhili, Nawal Mouhajir, Sara Bellefqih, Hanan Elkacemi, Tayeb Kebdani, Noureddine Benjaafar

**Affiliations:** 1 Service de Radiothérapie, Institut National d'Oncologie, Université Mohammed V Souissi, Rabat, Maroc

**Keywords:** ndomètre, cancer, chirurgie, radiothérapie, INO, Endometrium, cancer, surgery, radiotherapy, INO

## Abstract

Le cancer de l'endomètre est le cancer gynécologique le plus fréquent en occident. Il concerne principalement les femmes ménopausées. L'objectif de notre travail est de rapporter l'expérience du service de radiothérapie à l'Institut National d'Oncologie (INO) dans la prise en charge du cancer de l'endomètre. Nous avons analysé rétrospectivement 52 cas de cancer de l'endomètre traités dans le service de radiothérapie de l'INO entre 2007 à 2009. Les données collectées à partir des dossiers médicaux de nos patientes concernaient les aspects épidémiologiques, cliniques, thérapeutiques et évolutifs de ce cancer. La médiane d’âge des patientes était de 57 ans, 87% étaient ménopausées. Le délai moyen de consultation était de six mois. Le maitre symptôme était des métrorragies chez 51 patientes. Le diagnostic histologique a été porté sur un curetage biopsique de l'endomètre dans 51% des cas. L'examen anatomopathologique a montré un adénocarcinome endométrioïde dans 92% des cas. Après le bilan, 27% des patientes étaient stade I, 30% stade II, 20% stade III et 1% stade IVA selon la classification de la Fédération Internationale de Gynécologie Obstétrique (FIGO). Après la chirurgie, 51% des patientes ont reçu une radiothérapie externe. La dose délivrée était de 46 Gray (Gy). Une curiethérapie du fond vaginal a été délivrée chez toutes les patientes. Sur le plan évolutif, 83% des patientes étaient toujours suivies en situation de bon contrôle de leur maladie, 8% ont eu une récidive locorégionale et 4% avaient des métastases à distance. Ainsi, le cancer de l'endomètre est un cancer dont le traitement repose sur la chirurgie. La radiothérapie est le traitement adjuvant principal.

## Abstract (en)

Endometrial cancer is the most common gynecological cancer in the Western world. It affects mainly postmenopausal women. The aim of our study is to report the experience of the radiotherapy department of the National Oncology Institute (INO) in the treatment of endometrial cancer. We retrospectively analyzed 52 cases of endometrial cancer treated in the INO radiotherapy department between 2007-2009. Data obtained from the patient medical records were related to the epidemiologic, clinical, therapeutic and evolutionary aspects of this cancer. The median age of the patients was 57 years, 87% were postmenopausal. The median of consultation time was six months. The main symptom was metrorrhagia (51 patients). Histological diagnosis was based on biopsic curettage of uterine endometrium in 51% of the cases. Anatomo-pathological examination showed an endometrioid adenocarcinoma in 92% of the cases. After the assessment, 27% of the patients were stage I, 30% stage II, 20% stage III and 1% stage IVA, according to the International Federation of Gynecological and Obstetrics (FIGO) stage classification. After surgery, 51% of the patients received postoperative external radiotherapy. The delivered dose was 46 Gray (Gy). All patients received internal vaginal brachytherapy. From an evolutionary perspective, 83% of the patients had no recurrences during the follow-up period, 8% of patients had a local recurrence and 4% of patients had distant metastases. Therefore, surgery is the main treatment for endometrial cancer. Radiotherapy is the primary adjuvant treatment.

## Introduction

Le cancer de l'endomètre est le cancer gynécologique le plus fréquent en occident [1]. Il concerne principalement les femmes ménopausées [1]. La majorité des cancers de l'endomètre sont diagnostiqués à des stades précoces. Deux types histologiques du cancer de l'endomètre sont décrits: le type I et II, ayant des spécificités histologique, épidémiologique et moléculaire [2]. La chirurgie est le traitement de référence. Elle permet de préciser le stade selon la classification de la FIGO et ainsi de guider les indications du traitement adjuvant [3–5]. Son pronostic reste relativement favorable avec un taux de mortalité par cancer qui reste le plus faible en comparaison à des autres cancers féminins. L'objectif de notre travail est de rapporter l'expérience du service de radiothérapie à l'institut national d'oncologie dans la prise en charge du cancer de l'endomètre.

## Méthodes

Nous avons mené une étude rétrospective à travers une série de 52 cas du cancer de l'endomètre traités dans le service de radiothérapie à l'INO entre 2007 à 2009. Les données collectées à partir des dossiers médicaux de nos patientes en se basant sur une fiche d'exploitation, concernaient les aspects épidémiologiques, cliniques, thérapeutiques et évolutifs de ce cancer.

Le diagnostic était clinique et histologique. Les tumeurs étaient classées selon la classification de la FIGO; le bilan radiologique de l'extension locorégionale était une tomodensitométrie (TDM) abdomino-pelvienne, le bilan d'extension à distance était en fonction des signes d'appel. Le traitement reposait principalement sur la chirurgie qui permet de stadifier la tumeur et d'indiquer par la suite le traitement adjuvant: radiothérapie externe et/ou curiethérapie du fond vaginal. La chirurgie consistait à une hystérectomie totale voire une colpo-hystérectomie totale associée ou non à une annexectomie, associée ou non à une lymphadénectomie. La radiothérapie externe était délivrée par quatre faisceaux de photons X de haute énergie (18 à 25 MV). La dose totale délivrée à l´isocentre était de 46 Gy en 23 fractions, deux Gy par fraction.

La curiethérapie était de haut débit de dose (HDR), la dose totale délivrée variait en fonction de s'il s'agit d'une curiethérapie exclusive ou associée à une radiothérapie externe. Elle était soit 14 Gy en deux fractions hebdomadaires, sept Gy par fraction. Ou 24 Gy en quatre fractions hebdomadaires, six Gy par fraction.

## Résultats

Les caractéristiques des patientes sont résumées dans le Tableau 1. L’âge médian des patientes était de 57 ans (40– 76 ans), 87% étaient ménopausées. Le délai moyen de consultation était de 6 mois (2–36 mois).

**Tableau 1:** Caractéristiques des patientes

Caractéristiques des patientes	Nombre des patientes: N (%)
**Age**	
Moyenne d’âge	57 ans
Extrême d’âge:	40-76 ans
**Intervalle d’âge**	
40-50	8 (15%)
51-60	24 (46%)
61-70	15 (29%)
>71	5 (10%)
**Statut hormonal**	
Ménopausée	45 (87%)
Non ménopausée	7 13%)
**Parité**	
Multiparité	20 (38%)
Nulliparité	32 (62%)
**Antécédents (ATCD)**	
Diabète	18 (35%)
HTA	15 (29%)
HTA+ diabète	4 (8%)
Sans ATCD	19 (36%)

A la première consultation, 51 des patientes se plaignaient de métrorragies, une seule découverte fortuite lors d'un bilan de stérilité primaire. Le diagnostic histologique a été porté sur un curetage biopsique de l'endomètre chez 51 patients alors que chez une seule patiente la preuve histologique a été obtenue lors d'une hystérectomie totale pour un fibrome hémorragique, le Tableau 2 présente les différents aspects histologiques.

**Tableau 2:** Diagnostic histologique

Type histologique	Nombre des patientes: N (%)
Adénocarcinome endométroide:	48 (92%)
Grade 1	24 (50%)
Grade 2	18 (37%)
Grade 3	6 (13%)
Carcinome papillaire	2 (4%)
Carcinosarcome	1 (2%)
Carcinome mucineux	1 (2%)

Après le bilan, 27% des patientes étaient stade I, 30% de stade II et un % stade IVA selon la classification de la FIGO (Tableau 3).

**Tableau 3:** Stades de la tumeur

Stade	Nombre des patientes: N(%)
Stade I	34 (65%)
Stade II	11 (21%)
Stade III	6 (12%)
Stade IIIA	4 (7,7%)
Stade IIIB	1 (2%)
Stade IIIC	1 (2%)
Stade IV	1 (2%)

La chirurgie nous a permis de classer la tumeur et de déceler les facteurs pronostiques (Tableau 4) qui guident vers un traitement adjuvant, dont 51% des patientes ont reçu une radiothérapie externe. Une curiethérapie du fond vaginal de type HDR, a été délivrée à toutes les patientes. Une étude uni et multivariée de toutes les caractéristiques sus décrites a été réalisée dont seulement le type histologique reste indépendamment lié à la survenue d'une récidive tumorale avec un OR = 13,89 et c'est de façon statistiquement significative (p=0,034) (Tableau 5 et Tableau 6).

**Tableau 4:** Facteurs pronostiques chirurgicaux

Caractéristiques de la tumeur	Nombre des patientes: N(%)
**Type histologique**	
Adénocarcinome endométroide	48 (92%)
Autres	4 (8%)
**Taille de la tumeur**	
<2cm	10 (19%)
2,1 cm – 5 cm	23 (44%)
>5 cm	19 (37%)
**Marges d'exérèse**	
Saines	46 (88%)
Tumorales	6 (12%)
**Infiltration du myométre**	
≤ 50%	17 (33%)
>50%	35 (67%)
**Emboles vasculaires**	
Présentes	20(39%)
Absentes	22 (42%)
Non précisées	10 (19%)
**Envahissement ganglionnaire**	
Positif	6 (11,5%)
Négatif	40 (77%)
Non précisé	6 (11,5%)

**Tableau 5:** Analyse univariée des résultats

caractéristiques	OR	IC 95%	P
Age	1,003	[0,918-1,097]	0,94
Retard diagnostique	0,965	[0,868-1,073]	0,511
Geste chirurgicale	0,798	[0,3-2,1]	0,652
Type histologique	14,161	[1,223-163,93]	0,034
Taille tumorale	1,249	[0,728-2,144]	0,42
Grade histologique	1,525	[0,387-6]	0,546
Enboles vasculaires	2,4	[0,189-30,52]	0,5
Invasion du muométre	0,818	[0,147-4,557]	0,819
Structure envahis	1,744	[1,116-2,726]	0,015
Curage positif	5,25	[0,563-48,954]	0,145
Stade anapathologique	1,73	[1,149-2,603]	0,009

**Tableau 6:** Analyse multivariée des résultats

caractéristiques	OR	IC 95%	P
Type histologique	13,889	[1,137-169,671]	0,039
Structures envahies	0,924	[0,355-2,402]	0,871
Stade anatomopathologique	1,87	[0,837-4,177]	0,127

Sur le plan évolutif, après un recul moyen de 27 mois (1-55 mois), 83% des patientes étaient toujours suivies en situation de bon contrôle de leur maladie, 8% étaient atteintes de récidive locorégionale et 4% des métastases à distance (pulmonaire et cérébrale).

## Discussion

Les cancers de l'endomètre sont les cancers gynécologiques les plus fréquents en occident. Plus de 75% des patientes sont ménopausées au moment du diagnostic et seulement 3% ont moins de 40 ans [1], dans notre série 87% de nos patientes étaient ménopausées (Tableau 1). Parmi les facteurs de risque de ce cancer, on distingue principalement, l'obésité, le diabète et l'HTA, le traitement par tamoxifène [1, 2]. Les formes héréditaires représentent 2 à 5% des cancers de l'endomètre; On les observe principalement dans le syndrome de Lynch (cancer colorectal héréditaire sans polypose, cancer de l'endomètre, de l'estomac, de l'intestin grêle, pancréatique, ovarien, hépatobiliaire) [3], dans notre série aucun cas de forme héréditaire n'a été rapporté.

Deux formes cliniques et pronostiques sont actuellement décrites. Le carcinome endométroide de type 1 est d’évolution lente et de pronostic favorable. Le contexte est celui d'un état d'hyperœstrogénie et de surcharge pondérale. Il s'agit le plus souvent d'adénocarcinomes bien à moyennement différenciés. Cette forme de cancer de l'endomètre est souvent associée à des mutations génétiques (gènes K-ras, gènes RER) [2]. Le carcinome de type 2 se développe plus rapidement en dehors des facteurs de risque habituels (obésité, diabète, hyperœstrogénie). Sur le plan histologique, il s'agit de formes peu différenciées de type séreux ou à cellules claires. Cette deuxième forme de cancer de l'endomètre serait associée à des mutations des gènes p53 et/ou HER2 [2].

Le grade tumoral représente le degré de différenciation et influence considérablement sur le pronostic. Il s'agit le plus souvent d'un adénocarcinome endométrioïde. Les autres formes histologiques sont représentées par le carcinome mucineux, le carcinome à cellules claires, le carcinome papillaire séreux, le sarcome et le carcinosarcome; dans notre série leur taux est résumé dans le Tableau 2. Le carcinome à cellules claires et le carcinome papillaire séreux sont considérés comme de grade 3 et sont des formes d'emblée agressive. Les sarcomes représentent environ 5% des tumeurs malignes de l'utérus et comprennent les tumeurs mixtes du mésoderme, les léiomyosarcomes et les sarcomes de l'endomètre (stroma). Les sarcomes sont plus agressifs, provoquent plus fréquemment des métastases à distance [3].

Pour les circonstances de découverte, il s'agit essentiellement de métrorragies post-ménopausiques ou péri-ménopausiques, en général spontanées, indolores et peu abondantes. Les autres signes cliniques sont rares, il peut s'agir des leucorrhées, pesanteurs ou douleurs pelviennes, troubles urinaires. Dans notre série 98% de nos patientes, le signe clinique de découverte était des métrorragies. L'examen clinique est généralement peu informatif. En effet, l'examen du col utérin est le plus souvent normal sauf pour les stades II avec extension cervicale. L'exploration des aires ganglionnaires, la palpation du foie, la recherche d'une ascite, l'examen des seins sont systématiques [2].

Le bilan d'extension pré-thérapeutique comprend une hystéroscopie, une imagerie par résonance magnétique (IRM) abdomino-pelvienne qui est devenue actuellement le meilleur examen pour l’évaluation de la pénétration myométriale et l'invasion cervicale ou à défaut une TDM abdomino-pelvienne [4], dans notre série nos patientes on bénéficié d'une TDM abdomino-pelvienne.

La chirurgie est le traitement de référence d'un cancer de l'endomètre. Elle consiste en une hystérectomie totale avec salpingo-ovariectomie bilatérale. Les gestes supplémentaires sont la lymphadénectomie, l'omentectomie en fonction du stade clinique, du type histologique et du grade histologique [5]. La chirurgie permet de préciser le stade et d’établir les facteurs pronostiques [3–5].

La radiothérapie externe est réalisée suivant des modalités conformationnelles et selon les recommandations du Radiation therapy oncology group (RTOG), avec des photons de très haute énergie (au moins égale à 10 MV). Le volume d'irradiation dépend de l'extension tumorale. Elle se limite au pelvis, en l'absence d'atteinte ganglionnaire iliaque commune ou lomboaortique. En cas d'atteinte ganglionnaire lomboaortique, le volume d'irradiation inclut la région lomboaortique. La dose totale est de 45 à 50 Gy, avec 5 fractions hebdomadaires de 1,8 à 2 Gy. En cas d'irradiation exclusive, non précédée de chirurgie, une surimpression de ganglions suspects d'envahissement à l'imagerie peut être proposée jusqu’à une dose totale d'au minimum 60 Gy [6, 7].

La curiethérapie vaginale n'est plus utile à tous les stades de la maladie. La curiethérapie vaginale postopératoire est effectuée préférentiellement à haut débit de dose, évitant une hospitalisation et les complications de décubitus. Une dose de 21 à 24 Gy est délivrée en 3 séances de 7 Gy ou en 4 séances de 5 à 6 Gy, calculés à 5 mm d’épaisseur. En cas de curiethérapie pulsée ou à bas débit de dose, une dose de 50 Gy est délivrée, calculé à 5 mm d’épaisseur. Lorsque la curiethérapie HDR est effectuée en complément de la radiothérapie externe, une dose de 10 Gy est délivrée en 2 séances de 5 Gy, calculée à 5 mm d’épaisseur. En cas de curiethérapie pulsée ou à bas débit de dose, une dose de 15 Gy est délivrée, calculée à 5 mm d’épaisseur [8, 9].

La radiothérapie pelvienne améliore le taux de contrôle local pelvien de la maladie dans les formes de mauvais pronostic (stades II, grade 3, infiltration du myomètre supérieure à 50%). Elle n'a par contre pas d'impact sur l’évolution métastatique ni sur la survie [7].

La prise en charge des patientes atteintes de cancers de l'endomètre repose sur la chirurgie, qui permet d’établir le stade de la maladie selon la classification de la FIGO et de déceler les facteurs de mauvais pronostiques sur lesquelles la décision d'un traitement adjuvant est fondée, dont les plus reconnus sont: le stade, le grade histologique, le degré d'infiltration du myomètre, le type histologique, l’âge, l'infiltration endocervicale et la présence d'emboles tumoraux intra vasculaires [7]. Ainsi pour le stade I, on distingue trois groupes pronostiques [10].

Le groupe à faible risque comprend les adénocarcinomes endométroide sans envahissement myométrial ou avec un envahissement limité à moins de 50% du myomètre de grade 1 ou 2. Les études rétrospectives et un essai randomisé Suédois publié en 2009 ont tous confirmé que, bien que la curiethérapie de la voûte vaginale soit une thérapeutique bien tolérée, elle n'a pas d'impact significatif sur le contrôle local. Aucun traitement adjuvant ne peut donc être justifié pour ces patientes qui ont un risque de récidive vaginale faible, estimé à moins de 3%, ce d'autant que ces récidives sont accessibles à un traitement de rattrapage par irradiation [10], donc pour les cancers de stade IA et de grade 1 ou 2, aucun traitement complémentaire n'est donc recommandé.

Le groupe à risque intermédiaire est constitué des carcinomes de type I sans envahissement myométrial ou avec un envahissement limité à moins de 50% du myomètre (IA) de grade 3, et des carcinomes envahissant plus de 50% de l’épaisseur du myomètre (IB) de grades 1 et 2. La curiethérapie vaginale est le traitement adjuvant standard [10]. Quatre essais thérapeutiques ont démontré que chez les autres patientes du groupe, la radiothérapie pelvienne améliorait le taux de contrôle local pelvien de la maladie mais n'avait pas d'impact sur l’évolution métastatique ni sur la survie. Ceci a fait discuter l'intérêt de cette irradiation face à une curiethérapie seule potentiellement aussi efficace et moins toxique. Cette question a été posée par l'essai PORTEC 2 (Post Operative Radiation Therapy in Endometrial Carcinoma) 2. La présentation des résultats préliminaires à trois ans suggérait que les deux modalités thérapeutiques avaient une efficacité similaire en termes de survie sans récidive et de survie globale [11].

Le groupe à haut risque de récidive inclut les carcinomes de type I avec envahissement de plus de 50% de l’épaisseur du myomètre (IB) de grade 3 et les carcinomes de type II (IA et IB). Pour ces patientes, il est recommandé de faire une radiothérapie externe pelvienne et une curiethérapie de la voûte vaginale, qui ne permet cependant pas de réduire le risque de récidive à moins de 10%. Chez ces patientes, le taux d’évolution métastatique est aussi élevé, ce qui fait discuter la radio-chimiothérapie concomitante suivie d'une chimiothérapie adjuvante [12].

Dans le cas des tumeurs stades II: la conduite à tenir thérapeutique recommandée est une chirurgie suivie d'une radiothérapie avec ou sans curiethérapie. Dans les stades avancés (III et IV): les thérapeutiques doivent être plus agressives. On propose la chirurgie lorsqu'elle est possible car, associée à la radiothérapie, elle permet de meilleurs résultats qu'une irradiation exclusive. Dans les formes évoluées ou à haut risque de récidive, des essais incluant une chimiothérapie, soit exclusive, soit concomitante à une irradiation, ont été conduits ces dernières années. Les résultats de ces différents essais ont permis de montrer l'intérêt potentiel de la chimiothérapie pour diminuer [13].

## Conclusion

Le cancer de l'endomètre est un cancer dont le traitement repose sur la chirurgie. La radiothérapie est le traitement adjuvant principal. Ce cancer est généralement de bon pronostic. A travers cette étude rétrospective nous avons rapporté l'expérience de notre institut dans le traitement de cette tumeur.

### Etat des connaissance sur le sujet

- Le cancer de l'endomètre est le cancer gynécologique le plus fréquent en occident. Il concerne principalement les femmes ménopausées;
- La chirurgie est le traitement de référence. Elle permet de préciser le stade selon la classification de la FIGO. Et ainsi guider les indications du traitement adjuvant dont la radiothérapie avec ces deux volets (la radiothérapie externe et la curiethérapie du fond vaginal) représente une place principale.

### Contribution de notre étude a la connaissance

- L'objectif de notre travail est de rapporter les aspects épidémiologiques, diagnostiques du cancer de l'endomètre au service de radiothérapie à l'institut national d'oncologie;
- L'objectif de notre travail est de rapporter l'expérience du service de radiothérapie à l'institut national d'oncologie dans le traitement du cancer de l'endomètre;
- en analyse multivariée, et en ajustant sur le stade (FIGO) et les structures voisines envahies, seul le type histologique reste indépendemment lié à la survenue d'une récidive tumorale et ce de façcon statistiquement significative (p=0,034).
